# Development of a Staff Informant Measure of Lucidity

**DOI:** 10.1093/geroni/igab046.180

**Published:** 2021-12-17

**Authors:** Jeanne Teresi, José Luchsinger, Mildred Ramirez, Stephanie Silver, Davangere Devanand, Julie Ellis, Gabriel Boratgis, Paloma Gonzalez-Lopez

**Affiliations:** 1 Research Division, HHAR, Riverdale, New York, United States; 2 Columbia University, New York, New York, United States; 3 Research Division, Hebrew Home at Riverdale by RiverSpring Health, Riverdale, New York, United States; 4 Columbia University Irving Medical Center, New York, New York, United States; 5 La Trobe University, Nursing and Midwifery, Bundoora, Victoria, Australia

## Abstract

Lucidity Measure Development: An existing questionnaire measuring lucidity length, degree, content, coinciding circumstances, and time from lucid episode to death was expanded to include time of day, expressive and receptive communication and speech the month prior to and during the lucid event. Pilot Study: 33 interviews with staff were conducted; 73% reported ever witnessing paradoxical lucidity. Among 29 events reported, 31% lasted several days, 20.7%, 1 day, and 24.1% less. In 78.6% the patient engaged in unexpected activity. 20% died within 3 days and 17% within 3 months after the event. Qualitative Analyses: To refine the measure, 10 family caregivers and 20 LTSS staff caregivers completed a web-based focus-group type exercise using QualtricsXM. A content-thematic analysis with an inductive approach was applied to make qualitative inferences by analyzing the meaning and semantic relationship of words, phrases, and concepts. Using the reduction method of selection, conceptual content categories will be developed.

